# Bioactivity and dimensional stability of resin-, hybrid-, and silicate-based endodontic sealers: An in vitro comparative study

**DOI:** 10.4317/jced.63855

**Published:** 2026-04-25

**Authors:** Mirlyn de Souza Dias, Joana Lia Freitas Furtado, Maria Aline Ferreira Damasceno, Janaina Cabral de Carvalho, Delane Viana Gondim, Murilo Priori Alcalde, Marco Antônio Húngaro Duarte, Bruno Carvalho de Vasconcelos

**Affiliations:** 1 Postgraduate Program in Dentistry, Federal University of Ceará, Fortaleza, CE, Brazil; 2 Dentist, Federal Institute of Piauí, Teresina, PI, Brazil; 3 Department of Operative Dentistry, Endodontics, and Dental Materials — Bauru School of Dentistry, University of São Paulo

## Abstract

**Background:**

The success of endodontic treatment depends on selecting sealers with suitable physicochemical and biological properties. Solubility, pH, and calcium ion (Ca²+) release are critical factors influencing biocompatibility, antimicrobial activity, and long-term sealing.

**Material and Methods:**

This in vitro study compared three endodontic sealers, AH Plus (AHP), MTA Fillapex (MTAF), and EndoSequence BC Sealer (ESBC). pH was measured with a digital pH meter, and Ca²+ release was determined by atomic absorption spectrophotometry after 3, 24, 72, and 168 hours. Volumetric solubility was analyzed using micro-computed tomography before and after 7 days of immersion.

**Results:**

All sealers exhibited an alkaline pH. ESBC showed the highest pH and Ca²+ release at all time points, followed by MTAF. AHP released minimal and progressively decreasing calcium release. In volumetric analysis, ESBC demonstrated the greatest solubility, with pronounced volume reduction, whereas AHP and MTAF showed slight mass gain, indicating greater dimensional stability.

**Conclusions:**

Although ESBC exhibited higher bioactive potential, its excessive solubility may impair long-term sealing. AHP and MTAF presented more stable dimensional behavior, suggesting better long-term performance. These in vitro results highlight the balance between bioactivity and stability as a key factor when selecting root canal sealers for clinical use.

## Introduction

The success of endodontic treatment depends on achieving thorough disinfection and a hermetic, three-dimensional seal of the root canal system. Therefore, selecting sealers with appropriate physicochemical and biological properties is essential. Among these, solubility, pH, and ion release are fundamental characteristics, as they directly influence the biocompatibility, antimicrobial activity, and long-term stability of endodontic sealers (1 - 3). Sealer solubility is critical for maintaining the integrity of the obturation and preventing microleakage (4). Excessive solubility can compromise the apical seal, facilitating bacterial recontamination and, consequently, treatment failure. According to ADA and ISO 6876 standards (5), sealers should exhibit solubility below 3% to be considered clinically acceptable, ensuring an effective barrier against residual or newly introduced microorganisms (6). Inadequate sealing allows the passage of fluids and bacteria into the periapical tissues, thereby jeopardizing long-term treatment success (7 , 8). Beyond solubility, pH and ion release are important bioactive properties that influence the periapical environment and antimicrobial behavior (9). An alkaline pH helps reduce bacterial viability and prevent recolonization (10), while calcium ion release contributes to biomineralization and the formation of a mineral barrier, supporting tissue repair and periapical healing (6 , 11 - 13). Historically, Grossman's criteria (14) for ideal root canal sealers have guided material development, emphasizing easy handling, flowability, hermetic sealing, dimensional stability, antimicrobial activity, biocompatibility, radiopacity, and retrievability (15). Yet, no single material fulfills all these requirements. Among commonly used sealers, AH Plus (AHP), an epoxy resin-based material, is often regarded as the gold standard due to its low solubility, radiopacity, dimensional stability, and adhesion (1 , 2). However, it lacks bioactive potential such as ion release. To overcome these limitations, calcium silicate-based sealers like MTA Fillapex (MTAF) have been introduced, combining the bioactivity of mineral trioxide aggregate (MTA) with a resinous vehicle, providing high flow and an initially alkaline pH (2). More recently, bioceramic sealers such as EndoSequence BC Sealer (ESBC) have gained prominence for their calcium silicate composition (16 , 17), demonstrating dimensional stability and antimicrobial properties associated with their high pH (9 , 10 , 17). Despite these advances, few studies have directly compared the physicochemical and bioactive properties of these sealers under standardized conditions. Methodological variations across studies hinder accurate comparison and may bias clinical interpretation. Understanding how their distinct mechanisms of action translate into measurable properties is therefore crucial. In this context, the present in vitro study aimed to compare the pH, calcium ion release, and volumetric solubility of three endodontic sealers with distinct compositions: AHP (epoxy resin-based), MTAF (calcium silicate-resin hybrid), and ESBC (bioceramic). This comparative analysis seeks to identify the advantages and limitations of each formulation, providing data to support more informed clinical decisions and the development of next-generation endodontic biomaterials.

## Material and Methods

The root canal sealers evaluated in this study were AHP (Dentsply-De Trey GmbH, Konstanz, Germany), MTAF (Angelus, Londrina, Brazil), and ESBC (Brasseler USA Dental, Savannah, GA, USA). Their presentation forms, chemical compositions, and batch numbers, as provided by the manufacturers, are listed in Table 1.

Table 1

This in vitro study was prepared in accordance with the Preferred Reporting Items for Laboratory studies in Endodontology (PRILE) 2021 guidelines (18). - pH determination and calcium ion release Polyethylene tubes (internal diameter: 1.0 mm; length: 1.0 cm) sealed at one end were used for sample preparation. Each tube was completely filled with one of the sealers using Paiva pluggers and Lentulo spirals. Five specimens were prepared for each experimental group (n = 5). After filling, the specimens were immediately immersed in Falcon tubes containing 10 mL of deionized water and stored in an incubator at 37°C throughout the experimental period. All tubes were pretreated with nitric acid to avoid contamination, and negative controls (empty tubes; n = 3) were included for validation. Evaluations were performed after 3, 24, 72, and 168 hours. At each time point, the specimens were carefully transferred to new tubes containing the same volume of deionized water. The pH of each solution was measured using a calibrated digital pH meter (QM-400, Quimis, São Paulo, Brazil) with standard buffer solutions at pH 4.0, 7.0, and 10.0. After removing the specimens, the Falcon® tubes were placed in a tube shaker (Model 251; Farmem, São Paulo, Brazil) for 5 seconds. The solutions were then transferred to beakers, and pH readings were taken with the electrode. All values were recorded for subsequent analysis. For calcium ion release, the solutions collected at each time point were analyzed using an atomic absorption spectrophotometer (AA240FS; Varian, Santa Clara, USA). To minimize interference from phosphate and sulfate ions and to prevent the formation of refractory oxides, a strontium nitrate solution (10 g/L) was added. The instrument was operated at a wavelength of 422.7 nm, slit width 0.2 nm, lamp current 10 mA, and a slightly reducing stoichiometry, maintained by acetylene (2.0 L/min) and compressed air flow. - Volumetric solubility analysis For this analysis, acrylic resin teeth measuring 20 mm in length were used. Canals were instrumented with K3 rotary files (SybronEndo, Orange, USA) up to size #35/.06, with the working length set 1.0 mm short of the apex. Ten specimens (n = 10) were prepared for each group. For obturation, 0.06 taper gutta-percha cones (Odous de Deus, Belo Horizonte, Brazil) were used with all sealers (AH Plus, MTA Fillapex, and EndoSequence BC Sealer). Cones were calibrated to 350 µm using a measuring gauge (Dentsply-Maillefer, Ballaigues, Switzerland). Specimens were immersed in 50 mL of deionized water and stored in an incubator at 37°C for 7 days. Specimens were scanned before and after the experimental period using a micro-computed tomography (micro-CT) system (1174v2 SkyScan; Bruker-micro-CT, Kontich, Belgium) with acquisition parameters of 50 kV, 800 µA, 360° rotation, and isotropic resolution of 19.6 µm. Images (1304 × 1024 pixels) were reconstructed using NRecon software v.1.6.3 (Bruker-micro-CT) to generate transverse and axial sections. For qualitative evaluation, CTVol software v.2.2.1 (Bruker-microCT) was used to analyze area, perimeter, circularity, major and minor diameters, and aspect ratio. Quantification of obturation material volume was performed using CTAN software v.1.12 (Bruker-micro-CT), calculating the volume (mm³) in the apical and middle thirds of the canal, between 0.5 and 9.0 mm from the apex. - Statistical Analysis The normality of the data distribution was assessed using the Shapiro-Wilk test. All analyses indicated a non-parametric distribution. Associations were analyzed with the Kruskal-Wallis test, and when significant, Dunn's post hoc test was applied for multiple group comparisons. Data are presented as the median ± standard error of the mean (SEM). All statistical tests were performed using GraphPad Prism 8.0 (GraphPad Software, San Diego, CA, USA). Differences were considered statistically significant at p &lt; 0.05.

## Results

- pH evaluation All tested sealers exhibited alkaline pH values (Table 2). A general trend of decreasing alkalinity over time was observed, except for ESBC, which showed a progressive increase in pH during the initial periods, with mean values of 10.11, 10.56, and 10.79 at 3, 24, and 72 hours, respectively. Statistically significant differences were observed among the materials at all time points, with ESBC presenting the highest pH values across all evaluated intervals (Table 2).

Table 2

- Calcium ion release ESBC demonstrated significantly higher calcium ion release compared to the other sealers throughout all experimental periods (Table 3).

Table 3

In contrast, AHP exhibited a progressive decrease in calcium ion release over time, while MTAF showed a gradual increase in this parameter (Table 3). Values followed by different letters indicate statistically significant differences according to the Kruskal-Wallis test followed by Dunn's post hoc test (p &lt; 0.05). - Volumetric solubility All materials showed changes in volume during the experimental period. ESBC was the only material to present a median volume reduction (34.27%), which was statistically different from the other groups. Conversely, AHP and MTAF displayed slight increases in mass (Table 4).

Table 4

**Figure 1:**
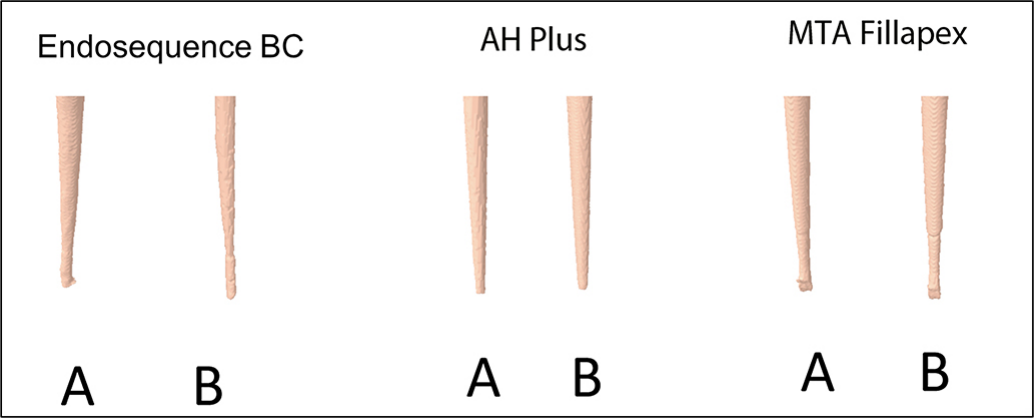
Representative micro-CT images of specimens from the experimental groups before (A) and after (B) the immersion period.

These quantitative results were supported by visual inspection of the specimens after immersion, confirming the volumetric changes (Fig. 1).

1

## Discussion

This study investigated key physicochemical properties of endodontic sealers that are directly related to their clinical performance, including alkalinizing potential, calcium ion release, and dimensional stability. Understanding these properties is essential because they influence the material's bioactivity, antimicrobial effects, and ability to maintain an effective root canal seal. The present findings provide insight into how different formulations behave under controlled conditions, highlighting the distinct characteristics of calcium silicate-based versus epoxy resin-based sealers and their potential implications for periapical healing and long-term treatment outcomes. Although bioactivity and ion release are desirable properties, dimensional stability and maintenance of the apical seal are more critical for long-term endodontic success. Our findings indicate that EndoSequence BC Sealer exhibits high alkalinity and calcium ion release but also greater volumetric solubility, which may compromise sealing integrity. Conversely, AH Plus and MTA Fillapex demonstrated superior volumetric stability, favoring durable obturation. The alkaline behavior observed for all tested sealers underscores their potential for bioactivity and antimicrobial action, consistent with previous reports (20 , 21). The sustained alkalinity of ESBC Sealer is particularly notable, reflecting continuous hydroxyl ion release from calcium silicate hydration, which may enhance mineralization and antimicrobial effects (22). In contrast, the transient pH peaks of AHP and MTAF appear to result from early dissolution of superficial particles during the setting phase, followed by stabilization of pH over time (20 , 21 , 23). These differences suggest that calcium silicate-based sealers may provide longer-lasting alkalinizing activity compared with epoxy resin-based materials, which could have implications for their biological performance and clinical outcomes. These reactions contribute not only to antimicrobial activity but also to the biological properties of these materials. Previous studies have demonstrated that the alkaline environment generated by calcium silicate-based materials can promote mineralized tissue formation and modulate cellular responses in the periapical region, favoring tissue repair processes (24). Calcium ion release is a key factor underlying the mineralization potential of endodontic sealers, as it contributes directly to hydroxyapatite formation and tissue repair (25). In agreement with previous reports, the limited ion release of AHP reflects the inert nature of epoxy resin-based materials, which exhibit minimal interaction with the surrounding fluids (26). In contrast, the higher and more sustained calcium ion release observed for MTAF and ESBC highlights the bioactive nature of calcium silicate-based formulations. The initial burst of ion release from these materials, particularly from ESBC, suggests rapid hydration and formation of calcium hydroxide, followed by a gradual stabilization over time. This mechanism, commonly described for bioceramic sealers, supports their potential to stimulate cellular signaling pathways involved in mineralization and tissue repair and to promote mineral deposition and periapical healing compared with resin-based materials (27). Recent studies have shown that these materials can enhance the adhesion, migration, and differentiation of human periodontal ligament stem cells, as well as promote the formation of mineralized nodules and the upregulation of osteogenic and cementogenic markers (28 , 29). Additionally, calcium silicate-based sealers may exert immunomodulatory effects by reducing the release of pro-inflammatory cytokines such as IL-6, thereby promoting a microenvironment favorable to periapical healing and regeneration (28 , 29). Micro-CT-based solubility analysis presents significant advantages over conventional methods described by ISO 6876 and ANSI/ADA No. 57 standards, which are predominantly based on mass loss after immersion (30). Traditional tests, although widely used, have considerable limitations such as the absence of three-dimensional analysis and great interference from factors such as water sorption and progressive material degradation over time, compromising the clinical representativeness of the results obtained (31 , 32). The high volumetric solubility observed for ESBC reflects the intrinsic behavior of calcium silicate-based formulations, in which hydrophilic nanometric particles promote fluid interaction and calcium hydroxide dissociation (33). While this ionic release underlies the material's bioactivity, it may compromise the integrity of the root canal filling, potentially increasing the risk of apical leakage. In contrast, the limited dimensional changes of AHP and MTAF confirm the greater stability of epoxy resin-based materials, aligning with previous studies demonstrating compliance with solubility standards (20 , 30 , 32). These findings emphasize a trade-off between bioactivity and structural stability that clinicians must consider when selecting root canal sealers. Overall, all tested sealers exhibited alkaline pH and calcium ion release, confirming their bioactive potential. ESBC demonstrated the highest ion release and alkalinity but also markedly greater volumetric solubility, which may compromise dimensional stability and sealing ability. In contrast, AHP and MTAF showed greater volumetric stability and lower ion release, suggesting more durable sealing over time. While ESBC presents promising biological properties, its high solubility warrants caution in clinical applications where dimensional stability is critical. Further in vivo and long-term studies are needed to clarify the clinical impact of these physicochemical differences and to support more precise recommendations for endodontic practice. The sealers tested showed some degree of bioactivity, but their clinical performance depends primarily on how well they maintain the root canal seal over time. AHP and MTAF presented lower bioactivity but superior volumetric stability, suggesting more predictable sealing over extended periods. For clinicians, this means that resin-based and hybrid sealers may offer greater reliability in maintaining obturation integrity, especially in cases where long-term stability is prioritized. Although ESBC has demonstrated properties that may support periapical healing, its high volumetric solubility raises concerns for long-term dimensional stability. In clinical situations where maintaining a stable and durable seal is essential, this level of solubility may be a limiting factor. It seems that its main indication would be for teeth affected by trauma or with endo-periodontal resorption or communications, as well as in treatments where material extravasation is inevitable. All these clinical conditions will be favored by an ion-rich environment and a cement that does not present difficulties in solubilization or dissociation.

## Figures and Tables

**Table 1:** Evaluated materials and their compositions.

Sealer	Manufacturer / Origin	Batch Number	Presentation Form	Main Composition (according to manufacturer)
AH Plus	Dentsply-De Trey GmbH, Konstanz, Germany	#1310000937	Two tubes (Paste A and Paste B) for manual mixing (3.0 mL each).	Paste A: Bisphenol-A Epoxy Resin, Bisphenol-F Epoxy Resin, Calcium Tungstate, Zirconium Oxide, Silica, Iron Oxide Pigments.
				Paste B: Dibenzylamine, Aminoadamantane, Tricyclodecane-diamine, Calcium Tungstate, Zirconium Oxide, Silica, Silicone Oil.
MTA Fillapex	Angelus, Londrina, Brazil	#31813	Base Paste Tube (18 g) and Catalyst Paste Tube (12 g) for manual mixing.	Base Paste: Salicylate Resin, Natural Resin, Calcium Tungstate, Nanoparticulated Silica, Pigments.
				Catalyst Paste: Diluent Resin, Mineral Trioxide Aggregate, Nanoparticulated Silica, Pigments.
Endosequence BC Sealer	Brasseler USA Dental, Savannah, GA, EUA	#12004SP	Pre-filled syringe (2.0 g), injectable, no mixing required.	Zirconium Oxide, Calcium Silicates, Monobasic Calcium Phosphate, Calcium Hydroxide, Fillers and Thickeners.

1

**Table 2:** pH values of endodontic sealers at 3, 24, 72, and 168 hours.

Sealer	3 hours	24 hours	72 hours	168 hours
AH Plus	8.87 (8.80 – 8.92)b	8.06 (8.05 – 8.08)b	7.86 (7.83 – 7.86)b	8.63 (8.63 – 8.67)b
MTA Fillapex	8.88 (8.72 – 9.12)b	8.85 (8.60 – 9.37)b	8.66 (8.11 – 9.04)ab	8.86 (8.01 – 9.02)b
Endosequence BC Sealer	10.11 (10.08 – 10.12)a	10.56 (10.55 – 10.63)a	10.79 (10.60 – 10.81)a	10.71 (10.58 – 10.77)a
Deionized water	7.0	7.0	7.0	7.0

Values are expressed as medians, with minimum and maximum values in parentheses. Values followed by different letters indicate statistically significant differences according to the Kruskal-Wallis test followed by Dunn’s post hoc test (p < 0.05).

**Table 3:** Calcium ion concentration (mg•L-¹) released from the sealers at 3, 24, 72, and 168 hours.

Sealer	3 hours	24 hours	72 hours	168 hours
AH Plus	0.75 (0.68 – 0.76)b	0.67 (0.63 – 0.69)b	0.48 (0.47 – 0.48)b	0.38 (0.37 – 0.40)b
MTA Fillapex	0.43 (0.43 – 0.44)b	1.33 (1.30 – 1.38)ab	1.45 (1.43 – 1.49)ab	1.61 (1.43 – 1.79)ab
Endosequence BC Sealer	5.64 (5.10 – 5.94)a	6.76 (6.64 – 6.89)a	5.73 (5.63 – 5.92)a	5.68 (5.46 – 5.90)a
Control	0.0	0.0	0.0	0.0

Values are expressed as medians, with minimum and maximum values in parentheses.

**Table 4:** Volumetric solubility (%) of endodontic sealers after 168 hours.

Sealer	168 hours
AH Plus	0.78 (-9.6 to 10.7)a
MTA Fillapex	0.22 (-12.83 to 15.76)a
Endosequence BC Sealer	-34.27 (-43.58 to -21.6)b

Values are expressed as medians, with minimum and maximum values in parentheses. Different letters indicate statistically significant differences according to the Kruskal–Wallis test followed by Dunn’s post hoc test (p < 0.05). Negative and positive values represent material loss and volumetric mass gain over the period, respectively.
